# Sequence, Structure, and Binding Analysis of Cyclodextrinase (TK1770) from *T. kodakarensis* (KOD1) Using an *In Silico* Approach

**DOI:** 10.1155/2015/179196

**Published:** 2015-12-24

**Authors:** Ramzan Ali, Muhammad Imtiaz Shafiq

**Affiliations:** Institute of Biochemistry and Biotechnology, University of the Punjab, Lahore 54590, Pakistan

## Abstract

Thermostable cyclodextrinase (Tk1770 CDase) from hyperthermophilic archaeon* Thermococcus kodakarensis* (KOD1) hydrolyzes cyclodextrins into linear dextrins. The sequence of Tk1770 CDase retrieved from UniProt was aligned with sequences of sixteen CD hydrolyzing enzymes and a phylogenetic tree was constructed using Bayesian inference. The homology model of Tk1770 CDase was constructed and optimized with Modeller v9.14 program. The model was validated with ProSA server and PROCHECK analysis. Four conserved regions and the catalytic triad consisting of Asp411, Glu437, and Asp502 of GH13 family were identified in catalytic site. Also an additional fifth conserved region downstream to the fourth region was also identified. The structure of Tk1770 CDase consists of an additional N′-domain and a helix-loop-helix motif that is conserved in all archaeal CD hydrolyzing enzymes. The N′-domain contains an extended loop region that forms a part of catalytic domain and plays an important role in stability and substrate binding. The docking of substrate into catalytic site revealed the interactions with different conserved residues involved in substrate binding and formation of enzyme-substrate complex.

## 1. Introduction

Enzymatic hydrolysis of polysaccharides is a method of choice in many industrial processes due to its high efficiency and better yields of the products as compared to acid hydrolysis. Glycoside hydrolases have been used for processing of starch, cellulose, hemicellulose, and cyclodextrins [1]. Cyclodextrins (CDs) are cyclic oligosaccharides with six or more glucopyranosyl units linked through *α*-1,4 glycosidic bonds. Cyclodextrins with six, seven, and eight glucopyranosyl moieties are termed as *α*-, *β*-, and *γ*-cyclodextrins, respectively. In water CDs adapt a structure with all hydrophilic groups directed towards exterior surface and hydrophobic groups towards an internal cavity. The internal hydrophobic cavity of CDs allows them to be resistant to hydrolysis by common amylases and also form inclusion complexes with different organic molecules [2, 3]. CDs have many applications in food, agriculture, cosmetics, and pharmaceutical industry. The vast applications of CDs and their hydrolytic products create a need for efficient and specific enzymes for CDs hydrolysis [2, 4, 5]. The glycoside hydrolases have been classified into 14 clans and 133 families. Each clan consists of at least two families that share catalytic fold and mechanism according to database of carbohydrate active enzymes [6]. The family GH13 (also called *α*-amylase superfamily), the largest family of glycoside hydrolases, is a member of clan GH-H along with families GH-70 and GH-77. The *α*-amylase family (GH13) is the most important family for industrial applications [7–9]. Although there is low sequence similarity among the enzymes of different families within this clan, they all exhibit certain structural features that have been conserved during evolution [10, 11].

The family GH13 is further classified into 35 subfamilies with at least 26 different specificities [12], including *α*-amylase (EC 3.2.1.1), pullulanase (EC 3.2.1.41), glucanotransferase (EC 2.4.1.25), and cyclodextrinase or cyclomaltodextrinase (EC 3.2.1.54). All of these subfamilies share a common catalytic domain comprising a TIM barrel or (*β*/*α*)_8_ barrel with a catalytic triad [13, 14] and a C-terminal domain consisting of *β*-strands only [10]. Many enzymes also possess N- and/or C-terminal carbohydrate binding modules like CBM34, CBM20, CBM41, and CBM48 [12, 15]. The CD hydrolyzing enzymes include cyclodextrinase, maltogenic amylase, and neopullulanase that hydrolyze CDs into linear maltodextrins or maltose [16, 17]. Recently thermostable pullulan hydrolase III from* Thermococcus kodakarensis* (KOD1) has also been reported to hydrolyze CDs into maltose or glucose [18]. The thermostable enzymes from hyperthermophiles have many advantages including higher rates of reaction, increased product yields, and decreased risks of contamination as compared to their mesophilic homologs [19–21]. Due to the advanced sequencing technologies and rapidly increasing numbers of genomes being sequenced, the number of sequences being classified as glycoside hydrolases is far exceeding the number of enzymes being structurally or biochemically characterized [22, 23]. Currently, GH13 family contains 26287 sequences but only 99 structures have been resolved [24]. Till the date of writing this work, Protein Data Bank contains only six CD hydrolyzing enzymes (PDB IDs: 4EAF, 1EA9, 2XIE, 1J0H, 1H3G, and 1BVZ) [6]. There is a need for better understanding of sequence and structural components of these proteins and their mechanism of catalysis as CDases. A bioinformatics approach can be used as a valuable predictive tool to provide information about structure and function of these enzymes.

In this work we have used an* in silico* approach to provide insight into the sequence, structural components, domain arrangement, catalytic machinery, and enzyme-substrate interactions of thermophilic cyclodextrinase (Tk1770) from* Thermococcus kodakarensis* (KOD1), an enzyme of potential industrial applications. This study provides the first attempt to use* in silico* approach to provide insight into the structure and key components of catalytic machinery of cyclodextrinase (CDase) from* T. kodakarensis.*

## 2. Materials and Methods

### 2.1. Sequence Retrieval, Alignment, and Phylogenetic Analysis

The amino acid sequence (UniProt ID Q5JJ59) of CDase from* T. kodakarensis* KOD1 (CDase-Tk; Tk1770) was retrieved from UniProtKB. A blast sequence similarity search was carried out against UniProtKB to find homologs of Tk1770. From the blast results sixteen different sequences of CD hydrolyzing enzymes from bacterial and archeal sources were selected for further studies (Table 1). The alignment of sequences was carried out with Clustal Omega and a rooted tree was generated using Bayesian inference method with default parameters [25, 26].

### 2.2. Homology Modeling

The Tk1770 CDase was subjected to NCBI BLAST against RCSB PDB (Protein Data Bank) to search suitable template(s) for comparative modeling. Multiple X-ray crystallographic structures (PDB ID: 4AEF, 1J0H, 4AEE, 1EA9, 1SMA, and 1WZL) with sequence identity from 56% to 29%, respectively, were selected as templates (Table 2). The sequences of target (Tk1770) and templates were aligned with Clustal Omega using UGENE program [25]. The alignment and the PDB structures were used as inputs for homology modeling with Modeller v.9.14 [27]. The model optimization was carried out by variable target function method (VTFM) with conjugate gradients (CG) and molecular dynamics (MD) with simulated annealing (SA) methods [27, 28]. The models generated by Modeller were scored on the basis of their DOPE (Discrete Optimized Protein Energy) values and the model with lowest DOPE score was selected for further studies. The homology model was further validated by ProSA-web server and PROCHECK [29, 30]. The model was refined by Modeller loop refinement functions and again validated for confidence. Thus, a reliable model was constructed and visualized using PyMOL [31].

### 2.3. Molecular Docking Studies

In order to investigate the enzyme-substrate interactions, the docking of substrates (*α*-, *β*-, and *γ*-cyclodextrins) into the active pocket of Tk1770 was carried out using AutoDock and MGL Tools v1.5.6 [33]. The substrates were prepared by adding polar hydrogen atoms and partial charges. The protein model was prepared by adding polar hydrogens and Gasteiger charges. The grid map dimensions were set around the active site with all other parameters set to default and rigid docking was performed. The candidates poses of the substrates were scored on the basis of their binding energy in kcal/mol and the best poses with lowest binding energy (kcal/mol) were selected.

## 3. Results and Discussion

### 3.1. Sequence Alignment and Phylogenetic Tree

**Figure 1:**
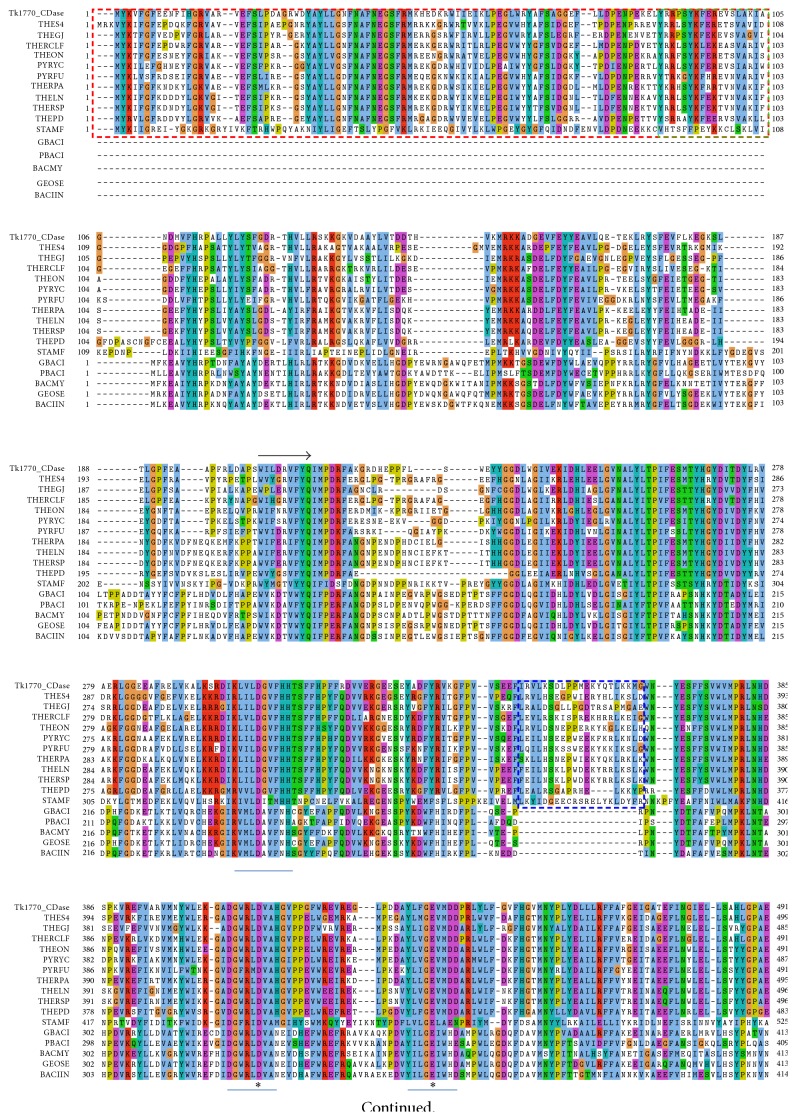
Sequence alignment of Tk1770 CDase with sixteen CD hydrolyzing enzymes. The alignment of Tk1770 CDase with archeal and bacterial CD hydrolyzing enzymes was carried out with Clustal Omega through UGENE package. The novel N′-domain (CBM48) in archeal sequences is represented in red and the protruding region of CBM48 domain in green dotted line. The arrow shows the start of the TIM barrel domain (residues 204–584) and four conserved regions (I–IV) with another downstream conserved region V are represented in grey line below sequence. The catalytic triad is indicated through esterics. The HLH region of archeal sequences that is absent in all bacterial homologs is represented in blue dotted line.

The sequence of Tk1770 consisting of 656 amino acids was aligned with sixteen CD hydrolyzing enzymes from the GH13 family (Figure 1). These sequences included eleven archeal enzymes and five bacterial enzymes having sequence identities from 28% to 60% with Tk1770 CDase (Table 1). All enzymes possess three major domains (i) an N-domain, (ii) a catalytic TIM barrel, and (iii) a C-domain [10, 34]. The sequence analysis showed that archeal enzymes contain two N-terminal domains (i.e., N′- and N-domain) in addition to the catalytic and C-domains, whereas the N′-domain is absent in all the bacterial CD hydrolyzing enzymes (Figure 1). A linker region from residues 190 to 203 in Tk1770 connects two N-terminal domains with two C-terminal domains. Four conserved regions of GH13 family in TIM barrel structure were identified from residues 299 to 310, 405 to 414, 433 to 441, and 496 to 502 with catalytic triad being Asp411, Glu437, and Asp502. An additional conserved region of amino acids 533–539 was also identified downstream to the conserved regions I–IV.

**Figure 2:**
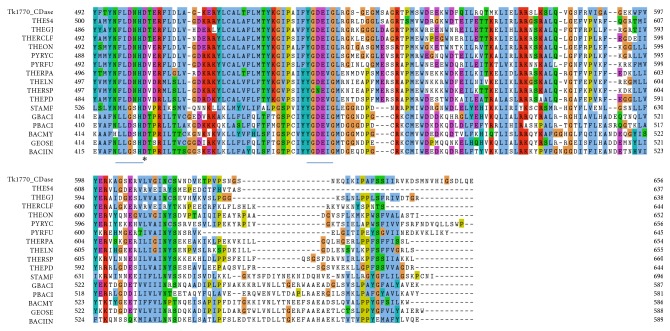
Phylogenetic tree: rooted radial tree of 17 CD hydrolyzing enzymes was constructed using MrBayes with Wag rate matrix (fixed) and visualized using FigTree. The phylogenetic tree obtained displays three distinct clades. All the bacterial enzymes form a single clade (shown in blue), while the branch for archeal enzymes split into two clades (shown in green and red). Depending upon sequence identity and domain arrangement Tk1770_CDase seems to be more closely related to THEGJ MAse, THES4 CDase, THERCLF CDase, PYRFU NPase, THEON CDase, and PYRYC CDase (green).

A rooted phylogenetic tree was constructed from alignment using MrBayes with rate matrix wag (fixed) to find evolutionary relationship. The tree was divided into three clades with all bacterial enzymes forming one clade and archeal enzymes divided into two clades (Figure 2). The tree showed that Tk1770 CDase is more closely related to THEGJ MAse and THES4 CDases with a sequence identity of 59% and 60%, respectively (Figure 2). The STAMF *α*-amylase shows 28% sequence identity with Tk1770 CDase and acts as outgroup in the phylogenetic tree. The *α*-amylases usually do not exhibit CD hydrolyzing activity and they also lack N′-domain. The *α*-amylase (STAMF *α*-amylase) from* S. marinus* is quite unique in this regard as it exhibits both CD hydrolyzing activity and additional N′-domain [35]. It suggests that during the course of evolution the presence of N′-domain might be linked to CD hydrolyzing activity in archaea.

### 3.2. Homology Modeling

The homology modeling program Modeller v9.14 [27] was used to construct 3D structure of Tk1770 with multiple templates as described in Materials and Methods. Out of five models generated the best model with lowest DOPE value was selected.

In homology modeling, sometimes the model might contain certain high-energy loops or residues with unusual geometry. Thus, the model selected was refined using Modeller built-in loop-refinement function on loops ranging from 3 to 7 amino acids in length and then validated with ProSA-web server and PROCHECK analysis [30]. The overall quality of the model was estimated by ProSA server in terms of *Z*-score by comparing it with *Z*-score values of experimentally resolved protein structures in Protein Data Bank [29]. Ramachandran plot validated all the nonglycine, nonproline residues to be in allowed regions and 87.9% of residues in most favorable regions. This verifies that all the residues exhibited accurate stereochemical positions.

**Figure 3:**
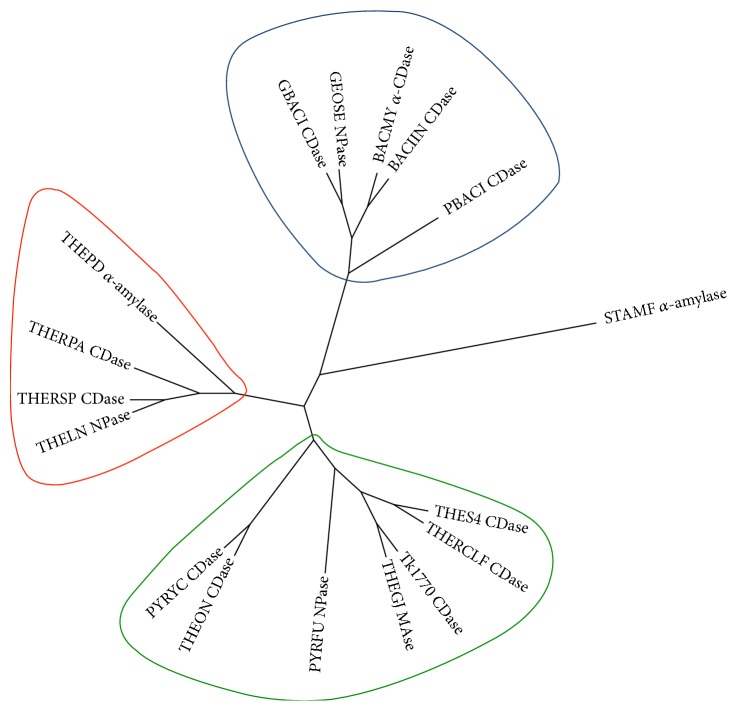
Structural features of Tk1770 CDase. (a) The schematic diagram representing the domain arrangement within Tk1770 made with software DOG (Domain Graph) v2.0 [32]. (b) The homology model of Tk1770 CDase consisting of N′- (blue), N- (yellow), catalytic (red), and C-domain (green). The catalytic domain also contains helix-loop-helix (HLH) structure (cyan). (c) Structural alignment of N′-domain (CBM48) of Tk1770 CDase (blue) model and template (4AEF) (grey) with an extension of loop into the catalytic site. The sequence alignment between the loops of model and template (4AEF) suggests that the substitution of P91 and S92 in Tk1770 makes its loop rigid.

Homology model of Tk1770 CDase was aligned with* P. furiosus *neopullulanase (PYRFU NPase) (PDB ID: 4AEF) for an analysis and comparison of the active site and other structural features. The overall structure of Tk1770 CDase folds into four major domains with two *β*-strands only N-terminal domains (i.e., N′- and conventional N-domain), connected to TIM barrel (A-domain) and a C-terminal domain, also consisting of *β*-strands. The structure of N′-domain of Tk1770 typically represents CBM48 with eight *β*-strands [15, 36]. The structural alignment of N′- or CBM48 domain of Tk1770 and PYRFU NPase revealed that both contain a loop that extends into the catalytic site. However, the extended loop of N′-domain of PYRFU NPase forms a more flexible helical turn as compared to the loop of Tk1770 (Figure 3). The substitution of P91 and S92 in extended loop region of Tk1770 in place of K89 and G90 in loop of PYRFU NPase might be responsible for this apparent decreased flexibility of loop in N′-domain of Tk1770 (Figure 3). Furthermore, K89 and G90 in extended loop of N′-domain in PYRFU NPase form strong hydrogen bonding with D460 and E470 of catalytic site. In Tk1770 S92 makes only one hydrogen bond with D460, thus reducing the interactions between the N′-domain and catalytic domain. Moreover, S92 in Tk1770 rotates backward to form hydrogen bond with Y93 that makes the loop more rigid. All of these factors may contribute to the decreased stability of the enzyme domains in Tk1770. Recently it was reported that the optimum temperature for Tk1770 CDase is 65°C, which is much lower as compared to optimal growth temperature for* T. kodakarensis* (85°C) and the optimum temperature for other archeal CD hydrolyzing enzymes [37].

The (*β*/*α*)_8_ barrel (A-domain) also contains a much larger B-domain between *β*-strand 3 and alpha-helix 3 from residues 306 to 403. The B-domain of all the archeal enzymes possesses a helix-loop-helix (HLH) motif that extends at the entrance of active site (Figure 3), but this HLH motif is absent in all five bacterial enzymes as shown in Figure 1. It has been reported that in order to maintain activity at high temperatures archaea might have adapted additional structural features. These features include N′-domain with a loop extension into the catalytic site and HLH motif that provides all necessary components for substrate binding and catalysis in a monomer [35, 38].

### 3.3. Docking of Substrates into the Catalytic Site

The docking of substrates into the catalytic site provided information about the interactions in enzyme-substrate complex. For this purpose, AutoDock was used to dock cyclodextrins, that is, *α*-, *β*-, and *γ*-cyclodextrins, into the active site of the Tk1770 CDase model. All of the conformations of ligands generated by the AutoDock were scored on the basis of their binding affinities in kcal/mol. The best poses of *α*-, *β*-, and *γ*-cyclodextrins were selected with binding energies of −8.8, −6.1, and −7.8 kcal/mol, respectively.

**Figure 4:**
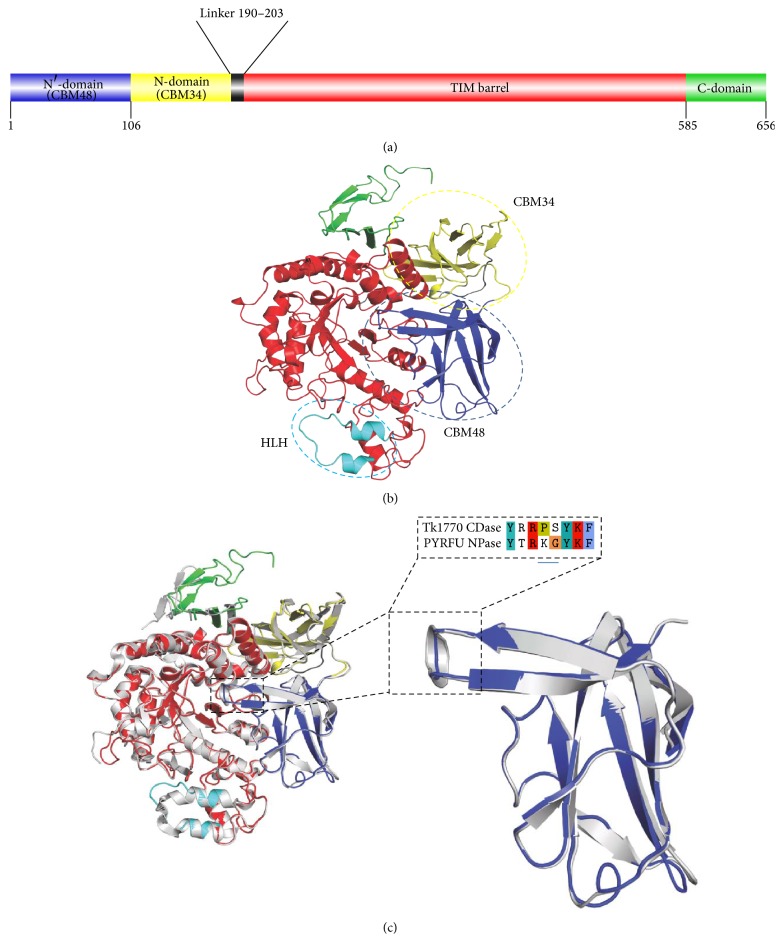
Docking of cyclodextrins into the active site of the Tk1770 CDase. (a) Complex of Tk1770 CDase with *α*-CD and residues involved in interactions. (b) The docked conformation and *β*-CD with TK1770 CDase. (c) The active site residues of Tk1770 interacting with *γ*-CD. The hydrogen bonds between substrate and amino acids are represented as red dashes.

The docking results showed that, apart from interacting residues, a number of residues come in close proximity with substrates, especially hydrophobic residues like Y93, F95, F373, F374, and V376. In case of docked *α*-cyclodextrin, residues D502, R550, and S375 formed hydrogen bonds with hydroxyl groups of substrate (Figure 4). In our homology model, K94 in the loop extension of N′-domain forms a salt bridge with E504 from the active site and might contribute to the stability of two domains, in the same manner as observed by Park et al. in amylase/neopullulanase (4AEF) from* P. furiosus* [38]. However, docking of *β*-CD showed strong interactions of K94 with hydroxyl groups of substrate and with E504 (Figure 4). Similarly, docking of *γ*-CD revealed interactions of K94, R97, and K364 with substrate (Figure 4). The amino acid K364 in helical region of HLH motif extends into the entrance of active pocket right above the F373 and F374 and might have a role in guiding the substrate into the active site. The aromatic amino acids Y88, Y93, and F95 that seem to be forming boundary wall of the active site and K94 protruding into the entrance of catalytic site are conserved in archeal homologs, except STAMF *α*-amylase.

## 4. Conclusion

Cyclodextrinase from hyperthermophilic archaea* T. kodakarensis *hydrolyzes cyclodextrins into linear maltodextrins. The sequence alignment of CD hydrolyzing enzymes confirmed that archaea have developed an additional N′-domain and a helix-loop-helix (HLH) motif in the B-domain that is absent in all bacterial homologs. The homology model constructed revealed that loop connecting *β*-strand 7 and *β*-strand 8 of N′-domain extends into the catalytic site (A-domain) and plays an important role in substrate binding. Residues Y88, Y93, K94, F95, and R97 in extended loop of N′-domain of Tk1770 CDase are conserved in CD hydrolyzing enzymes of archaea. Structural alignment between model and template (4AEF) indicated that P91 and S92 in loop extension of N′-domain of Tk1770 might decrease its flexibility and interactions with A-domain. This might contribute to the decreased stability of two domains in Tk1770.

The docking studies indicated that residues K94, R97, K364, S375, D502, E504, and R550 form hydrogen bonds with substrates. Residue K364 in the helix of HLH motif extending at the entrance of the catalytic site interacts with substrate and might be involved in guiding the substrate into the catalytic site. From these results it can be inferred that archeal CD hydrolyzing enzymes have developed catalytic machinery in which an extension of N′-domain not only constitutes a part of active pocket, but also plays an important role in substrate binding.

## Figures and Tables

**Table 1:** List of the sequences used for alignment and phylogenetics.

Enzyme	Organism	Abbreviation	A.A	Seq. similarity to Tk1770	UniProt ID
Cyclomaltodextrinase	*T. kodakarensis*	Tk1770 CDase	656	100%	Q5JJ59
Cyclomaltodextrinase	*Thermococcus *sp. (*strain CGMCC*)	THES4 CDase	637	60%	G0HJP6
Maltogenic *α*-amylase	*T. gammatolerans*	THEGJ MAse	638	59%	C5A4D9
Cyclomaltodextrinase	*T. cleftensis*	THERCLF CDase	644	59%	I3ZTQ5
Cyclomaltodextrinase	*T. onnurineus*	THEON CDase	652	59%	B6YV58
Cyclomaltodextrinase	*Pyrococcus yayanosii*	PYRYC CDase	656	57%	F8AHJ5
Neopullulanase	*Pyrococcus furiosus*	PYRFU NPase	645	56%	Q8TZP8
Cyclomaltodextrinase	*T. paralvinella*	THERPA CDase	654	56%	W0I4Q4
Neopullulanase	*T. litoralis*	THELN NPase	655	55%	H3ZKI8
Cyclomaltodextrinase	*Thermococcus *sp.* B1001*	THERSP CDase	660	54%	Q9HHC8
*α*-amylase	*T. pendens*	THEPD *α*-amylase	644	52%	A1S075
*α*-amylase	*Staphylothermus marinus*	STAMF *α*-amylase	696	28%	A3DM60

Cyclomaltodextrinase	*Geobacillus *sp. *G1w1*	GBACI CDase	587	32%	A0A093UHG3
Cyclomaltodextrinase	*Paenibacillus wynnii*	PBACI CDase	581	31%	A0A098M8Z8
*α*-cyclomaltodextrinase	*Bacillus mycoides*	BACMY *α*-CDase	586	30%	C3APY4
Neopullulanase	*G. stearothermophilus*	GEOSE NPase	588	31%	Q9AIV2
Cyclomaltodextrinase	*Bacillus indicus*	BACIIN CDase	589	29%	A0A084GIJ0

A.A means amino acids.

**Table 2:** List of the PDB files used as templates for homology modeling of CDase_Tk1770.

Serial number	PDB ID	Organism	Enzyme	% identity with TK1770	% query cover
1	4AEF	*P. furiosus*	Amylase	56	98
2	1EA9	*Bacillus *sp.	Cyclomaltodextrinase	33	76
3	1J0J	*G. stearothermophilus*	Neopullulanase	33	78
4	1SMA	*Thermus *sp.	Maltogenic amylase	32	79
5	4AEE	*S. marinus*	Maltogenic amylase	29	95
6	1WZL	*Thermoactinomyces vulgaris*	*α*-amylase II	35	76
